# *MiR-425-5p* suppression of *Crebzf* regulates oocyte aging via chromatin modification

**DOI:** 10.1007/s11357-023-00875-6

**Published:** 2023-08-03

**Authors:** Kadiliya Jueraitetibaike, Ting Tang, Rujun Ma, Shanmeizi Zhao, Ronghua Wu, Yang Yang, Xuan Huang, Xi Cheng, Cheng Zhou, Hong Zhang, Lu Zheng, Xie Ge, Li Chen, Bing Yao

**Affiliations:** 1 Department of Reproductive Medicine, Nanjing Jinling Hospital: East Region Military Command General Hospital, Medical School of Nanjing University, Nanjing, 210002 People’s Republic of China; 2 State Key Laboratory of Reproductive Medicine, Nanjing Medical University, Nanjing, 211166 People’s Republic of China; 3 Jiangsu Key Laboratory for Molecular and Medical Biotechnology, College of Life Sciences, Nanjing Normal University, Nanjing, 210002 People’s Republic of China; 4 Basic Medical Laboratory, Institute of Clinical Laboratory Medicine, Affiliated Jinling Hospital, Medical School of Nanjing University, Nanjing, 210002 People’s Republic of China

**Keywords:** *Crebzf* / H3K4me3, *miR-425-5p*, Oocyte aging, Transcriptional silence

## Abstract

Female infertility due to declining oocyte quality with age remains a significant challenge for patients and physicians, despite extensive research efforts. Recent studies suggest that microRNAs (miRNAs), which respond to various stressors in the aging process, may provide a promising solution. With the approval of small RNA drugs for clinical use, miRNA-based treatment of oocyte aging appears to be a viable option. Through high-throughput sequencing, *miR-425-5p* was identified as the only miRNA elevated under natural aging and oxidative stress. Microinjection of inhibitors to inhibit *miR-425-5p* effectively improved compromised phenotypes of old oocytes in vitro. Further investigation revealed that *Crebzf* acts as a mediator of *miR-425-5p*'s age-related functions in old oocytes. In vivo treatment with *miR-425-5p* antagomirs significantly improved impaired oocyte development in reproductively old females by targeting *Crebzf*. Single-cell RNA sequencing revealed that *Crebzf* plays a vital role in regulating mRNAs targeting histone H3, trimethylated lysine 4 (H3K4me3), a crucial marker for transcriptional silencing. Overexpression of *miR-425-5p* could hinder oocyte maturation by downregulating *Crebzf* expression and disrupting transcriptional regulation. Our findings provide new insights into the potential of *miR-425-5p* antagomirs as a treatment for female infertility and highlight an elegant mechanism by which *miR-425-5p* inhibition of *Crebzf* inhibits a developmental switch in GV oocytes by regulating a group of histone methyltransferase mRNAs.

**Supplementary Information:**

The online version contains supplementary material available at 10.1007/s11357-023-00875-6.

## Introduction

Delayed conception in women can lead to age-related fertility decline, which poses a challenge for both patients and physicians. Current scientific knowledge suggests that the primary causes of age-related infertility are decreased ovarian reserve and lower developmental competence of oocytes [1, 2]. Oocyte depletion is an irreversible biological process, making quality control of oocyte aging the only viable target for treatment. Despite decades of research, the currently available remedies to improve oocyte aging are far from satisfactory [3, 4].

During antral follicle maturation, germinal vesicle (GV) oocytes undergo chromatin structural changes from non-surrounded nucleolus (NSN) to surrounded nucleolus (SN) in response to gonadotropins, while they experience minimal growth in size [5, 6]. Accumulating evidence suggests that these chromatin modifications are necessary for the final growth of GV oocytes to prepare for meiotic maturation following an luteinizing hormone (LH) surge in vivo [7, 8]. Therefore, gaining a deeper understanding of the dynamic changes in chromatin structure and epigenetic modifications, such as noncoding RNAs, DNA methylation, and histone modifications in GV oocytes, could pave the way for developing novel therapeutic options.

One suggested link between aging and infertility is an imbalance between the generation and removal of reactive oxygen species (ROS), leading to oxidative stress (OS) [9]. In oocytes, accumulated ROS levels are closely associated with the aging process, leading to genetic and epigenetic modifications that impair oocyte maturation [10]. In reproductively older females, OS-induced mitochondrial dysfunction is critical for oocyte meiotic disorder, as has been well established [11, 12]. Moreover, several lines of evidence indicate that OS-mediated changes in chromatin structure and histone posttranslational modifications are involved in age-related processes [13].

Recent research has identified microRNAs (miRNAs) as a response mechanism to stress-induced cellular events [14]. Specifically, a recent study demonstrated that *miR-425-5p* functions as a modulator in abnormal energy metabolism induced by cold stress [15]. *MiR-425-5p* has been found to regulate cell proliferation and migration by targeting downstream mRNAs in various cell lines [16, 17]. Although *miR-425-5p* has been detected in oocytes and other germ cells [18, 19], its role in oocyte maturation had not been previously investigated.

The use of miRNA-based treatments in diseases such as cancer and chronic heart failure has sparked interest in exploring new therapeutic options for infertility treatment. Our study discovered higher levels of *miR-425-5p* in GV oocytes harvested from reproductively aged females, which was linked to poor oocyte quality. As a result, we proposed in vitro and in vivo therapeutic strategies that involve inhibiting *miR-425-5p*, which resulted in the restoration of oocyte quality in both cases. Additionally, we demonstrated an intricate mechanism through which *miR-425-5p* overexpression targeted *Crebzf* in mammalian GV oocytes, disrupting chromatin modification and transcriptional silencing. GV oocytes that overexpressed *miR-425-5p* or lacked *Crebzf* failed to accumulate histone H3, trimethylated lysine 4 (H3K4me3) and achieve transcriptional silencing. Our findings suggest that *miR-425-5p* regulates *Crebzf*, which interferes with chromatin modification and transcriptional silencing in GV oocytes, making it a potential therapeutic target for improving the developmental competence of oocytes.

## Materials and methods

### Mice

All animal experiments were performed following the rules and guidelines of the local animal ethics committee and the Animal Care and Use Committee of Nanjing Jinling Hospital (2020JLHGKJDWLS-175). Female Institute of Cancer Research (ICR) mice were purchased from Beijing Vital River Laboratory Animal Technology Co., Ltd. (Nanjing, China) and were housed in ventilated cages in a 12-h light:12‐h dark cycle at constant temperature (22 °C) and under controlled humidity. 4-week-old female mice were used as young controls and 10- to 12-month-old female mice near the end of their reproductive lifespan were used in experiments to generate a natural aging mouse model.

### Oocyte collection and culture

4-week-old and 10- to 12-month-old female ICR mice were used to collect oocytes. The estrous cycles of experimental mice were observed and identified using vaginal smears [20]. Following the continuous monitoring of two consecutive cycles, mice with typical estrous patterns were chosen for the experiment. Euthanasia of the mice was performed on the day of estrus detection by carbon dioxide asphyxiation followed by cervical dislocation. Mouse ovaries were first digested in the M2 medium with 1.5 μM milrinone (Sigma‒Aldrich, Burlington, MA, United States), and GV oocytes were isolated by gently puncturing small antral follicles (200–300 μm) with a sterile syringe needle under the visual field of a stereomicroscope (Nikon instrument, Amsterdam, Netherlands). Immature GV (GV0) oocytes were placed in the M2 medium supplemented with milrinone and cultured for 16 h to grow into fully grown GV (GV1) oocytes. GV1 oocytes were then washed, moved to M2 medium, and cultured for 3 h to evaluate the ratio of germinal vesicle breakdown (GVBD) and 14 h to evaluate the ratio of polar body exclusion (PBE). For the immunofluorescence analysis described below, GV1 oocytes were cultured in the M2 medium for 9 h to reach the metaphase I (MI) stage and 16 h to reach the metaphase II (MII) stage.

### Human Controlled ovarian hyperstimulation and follicular fluid collection

Follicular fluid were collected from 20 patients (aged from 20 to 35, grouped as young) and 20 patients (aged from 38 to 52, grouped as advanced maternal age) who underwent Intracytoplasmic sperm injection (ICSI) treatment at our clinic. All the procedures were approved by the Institutional Ethics Committee of Nanjing Jinling Hospital (2020DZGZRZX-088). All participants gave their written informed consents. The primary indication criterion for ICSI was male factor. Exclusion criteria were as follows: women with body mass index (BMI) < 18 or > 24, chronic hypertension, heart disease, diabetes, or donor oocyte/embryo recipient cycles.

All patients were stimulated with full-dose early follicular phase downregulation protocols with slight modifications. Patients received an injection of 3.75 mg of long-acting triptorelin acetate (Dipherelin IPSEN, France) on the 2^nd^-4^th^ day of the menstrual cycle and underwent endocrinology and ultrasound examinations 35–42 days after that. Suppose complete pituitary downregulation was achieved (endometrial thickness ≤ 5 mm, basal follicle-stimulating hormone (FSH) ≤ 5 mIU/mL, LH ≤ 5 mIU/mL, estradiol (E2) ≤ 50 pg/mL) and the diameter of the follicles were between 4 and 6 mm under ultrasound, supplementation with recombinant FSH (Gonal-F®, Merck, Switzerland) was given according to the participant's BMI (18 ≤ BMI ≤ 22, starting dose 112.5 IU/d; 22 < BMI ≤ 24, starting dose 150 IU/d). An injection of 10,000 IU of human chorionic gonadotropin (hCG; Livzon; Beijing, China) was administered intramuscularly when three or more leading follicles reached a diameter of 18 ± 20 mm. Oocyte retrieval was performed by transvaginal aspiration 36 h after hCG administration.

In each patient, the fluid volume was recorded, and the follicular fluid from the most mature follicle (18 ± 20 mm diameter) was individually aspirated. After removal of the oocytes, samples were centrifuged at 3 000 × g for 10 min to remove debris, blood, and granulosa cells as previously described [21]. Then, the follicular fluid supernatant was transferred to sterile polypropylene tubes and used for further assays.

### Hydrogen peroxide (H_2_O_2_) assay

H_2_O_2_ levels in follicular fluid were measured using a H_2_O_2_ assay kit (Abcam, Cambridge, United Kingdom) according to the instructions supplied by the manufacturer. Briefly, follicular fluid samples were centrifuged for 15 min at 1000 × g within 30 min of collection to remove particulate pellets and maintained on ice for further deproteinization. The absorbance values were detected with a Multimode Microplate Reader (BioTek Instruments, Winooski, Vermont, United States). The H_2_O_2_ concentration of each sample was calculated on the basis of the standard curve.

### In vivo experiments

Three 4-week-old female mice were injected with negative control (NC) (Ribobio Technology, Guangzhou, China). Nine 10-to 12-month-old female mice were randomly assigned to three groups. Mice in each group received injections of either *miR-425-5p* antagomirs (Ribobio Technology, Guangzhou, China) at a medium dose of 50 mg/kg ( +) or a high dose of 80 mg/kg (+ +), or NC [22, 23]. All mice received intraperitoneal injections on three consecutive days and were euthanized on the morning of day 7.

### Single-cell library preparation

In each of the three independent experiments, immature GV oocytes were collected from 3 mice. The oocytes were then divided into two groups: one group was microinjected with NC small interfering RNA (siRNA), while the other group received *Crebzf* siRNA microinjection. Following microinjection, the oocytes were cultured in drops of M2 medium containing 1.5 μM milrinone at 37 °C with 5% CO_2_ for a duration of 16 h. After the culture period, we collected 10 oocytes from each group and transferred them to a nuclease-free 0.2-mL PCR tube containing lysis buffer. The PCR tubes were marked and immediately placed on dry ice to preserve the samples. Overall, we analyzed a total of 30 oocytes from the control group and 30 oocytes treated with *Crebzf* siRNA. Then, the samples were incubated at 72 °C and immediately placed back on the ice, followed by spinning down and reverse transcription to cDNA based on the polyA tail. The template at the 5' end of the RNA was switched, and the full-length cDNA was amplified by PCR. The average molecule length was determined using an Agilent 2100 bioanalyzer instrument (Agilent, Beijing, China). PCR products were purified and selected with an Agencourt AMPure XP-Medium kit (Beckman Coulter, Brea, California, United States). The double-stranded PCR products were heat denatured and circularized by a splint oligo sequence. The single-strand circular DNA was formatted as the final library. The library was qualified by an Agilent Technologies 2100 bioanalyzer and amplified to make DNA nanoballs (DNBs) containing more than 300 copies of one molecule. The DNBs were loaded into the patterned nanoarray, and single-end 50-base reads were generated by combinatorial probe-anchor synthesis.

### RNA sequencing and data analysis

The sequencing data were filtered with SOAPnuke (v1.5.2) [24] by (1) removing reads containing sequencing adapters; (2) removing reads whose low-quality base ratio (a base quality less than or equal to 5) was more than 20%; and (3) removing reads whose unknown base ('N' base) ratio was more than 5%. Next, clean reads were obtained and stored in FASTQ format. The clean reads were mapped to the reference genome using HISAT2 (v2.0.4) [25]. Then, EricScript (v0.5.5) [26] and rMATS (v3.2.5) [27] were used to identify fusion and differentially spliced genes, respectively. Bowtie2 (v2.2.5) [28] was applied to align the clean reads to the gene set, which was generated by Beijing Genomic Institute (Shenzhen, China). Then, the expression level of genes was calculated by RSEM (v1.2.12), and a heatmap was drawn by pheatmap (v1.0.8) according to the gene expression in different samples. Essentially, differential expression analysis was performed using DESeq2 (v1.4.5) with a Q value ≤ 0.05. To gain insight into the change in phenotype, enriched Gene Ontology (GO) terms with a *p* value < 0.05 were obtained from the Database for Annotation, Visualization, and Integrated Discovery.

### RNA isolation and qPCR analysis

Total RNA from oocytes was extracted using an Arcturus™ PicoPure™ RNA isolation kit (Thermo Fisher Scientific, Waltham, Massachusetts, United States) according to the manufacturer's instructions. Quantitative RT‒PCR of the mRNA was performed using PrimeScript™ RT Master Mix (TaKaRa, Kusatsu, Shiga, Japan) and AceQ qPCR SYBR Green Master Mix (Vazyme). Relative mRNA levels were calculated by normalizing endogenous *Gapdh* mRNA levels (used as the internal control). The primers used are listed in Supplementary Table 1. RT‒qPCR analysis of miRNAs was performed with a miDETECT A TRACK™ miRNA qRT‒PCR Starter kit (RiboBio). The miRNA primers or RT‒qPCR were synthesized by RiboBio, and the primer sequences were commercially restricted. The expression of miRNA was normalized to the expression of the small nuclear RNA U6, the endogenous control.

### Run-on transcription assays

Transcriptional activity was determined in run-on studies with 5-Ethynyl-uridine (5- EU) using a Click-iT RNA Alexa Fluor 488 imaging kit (Thermo Fisher Scientific). The oocytes were cultured in an M2 medium with or without 2 mM EU at 37 °C for 45 min. After incubation with EU, the oocytes were briefly transferred to acid Tyrode's solution (Sigma‒Aldrich) to remove the zona pellucida and washed three times in phosphate-buffered saline (PBS, Genom, Zhejiang, China). The oocytes were then immediately fixed in 4% paraformaldehyde (PFA, Biohao Biotechnology, Guangzhou, China) for 30 min and washed three times with PBS as described above. The oocytes were then permeabilized with 0.1% Triton X-100 (Sigma‒Aldrich) in PBS for 1 h at room temperature and washed 3 times in PBS. The oocytes were then incubated for 30 min at room temperature with the Click-iT reaction cocktail prepared per the manufacturer's protocol and washed 3 times again with PBS. DNA staining was performed using Hoechst 33,342 (Beyotime, Shanghai, China) at 1:1000 in PBS for 15 min immediately before imaging. The fluorescence signal was detected using the 40 × objective of an LSM 710 laser scanning confocal microscope (Carl Zeiss, Oberkochen, Germany).

### ROS evaluation

A ROS assay kit (Beyotime) was used to detect intercellular ROS in living oocytes. According to the manufacturer's instructions, CM‐H2DCFDA was prepared in dimethyl sulfoxide (DMSO) before loading. Oocytes were incubated with 5 μM CM‐H2DCFDA for 30 min at 37 °C and then immediately observed under an IX73 inverted microscope (Olympus, Tokyo, Japan).

### Western blotting

Ovarian extracts were prepared from ovarian samples using cell lysis buffer (Beyotime) supplemented with protease and phosphatase inhibitor cocktail (Roche Diagnostics, Basel, Switzerland). After the transient ultrasound, the ovary lysates were incubated on ice for 30 min and then centrifuged at 4 °C and 12,000 × rpm for 20 min. The supernatant was transferred to a new tube, and an equal volume of loading buffer was added. For oocyte samples, a pool of 100 oocytes was lysed in 2 × Laemmli sample buffer (Bio-Rad, Hercules, California, United States) with protease inhibitor. After boiling at 95 °C for 10 min, the protein lysates were used for immunoblot analysis. Samples were separated on 10% sodium dodecyl-sulfate (SDS) polyacrylamide gel and transferred to polyvinylidene difluoride (PVDF) membranes. The membranes were blocked with bovine serum albumin (BSA, Biofroxx, Einhausen, Germany) in Tris-buffered saline with 0.1% Tween 20 detergent ( TBST, 10 mM Tris, pH 7.5; 150 mM NaCl; and 0.1% Tween-20) and then probed with primary antibodies overnight at 4 °C. The following primary antibodies at the stated dilutions used were anti-CREBZF (1:500, Abcam); anti-GAPDH (1:20,000, Proteintech, Rosemont, United States); anti-H3K4me3 (1:1,000, Abcam) and anti-histone H3 (1:2,500, Millipore, Burlington, Massachusetts, United States). After multiple washes with TBST and incubation with horseradish peroxidase (HRP)-conjugated secondary antibodies (Beyotime), the protein bands were visualized using an automatic chemiluminescence image analysis system (Tanon Science & Technology, Shanghai, China).

### Immunofluorescence and image processing

Immunofluorescence was performed as described previously [29]. In brief, oocytes were fixed with 4% PFA and permeabilized with 0.5% Triton X-100 before blocking with BSA in PBS. Samples were incubated overnight at 4 °C with primary antibodies and fluorescein isothiocyanate (FITC)-conjugated tubulin antibody (1:200, Sigma‒Aldrich). The following primary antibodies at the stated dilutions used were anti-CREBZF (1:100, Affinity Biosciences, Melbourne, Victoria, Australia), anti-H3K4me3 (1:100, Abcam), and anti-histone H3 (1:2,000, Proteintech). After multiple washes with PBS and incubation with Immunoglobulin G (IgG) / tetramethylrhodamine (TRITC) secondary antibodies (1:200, OriGene, Rockville, Maryland, United States), Hoechst 33,342 (1:500, Beyotime) was used to stain the chromosomes. After multiple washes in PBS, the oocytes were mounted and examined under an laser scanning microscope 710 (LSM 710). ImageJ software (version 1.52a, National Institutes of Health) was used to assess the fluorescence intensity. Fluorescence intensity = Integrated density – (Area of selected region X Mean fluorescence of background readings) [30].

### Microinjection of oocytes

SiRNAs targeting *Crebzf* and NC (Si-*Crebzf* #1, #2, and #3 and Si-NC) were designed and synthesized by RiboBio. For *Crebzf* cRNA synthesis, the PEXP-RB-Mam-*Crebzf* recombinant plasmid (RiboBio) was linearized by Not I, and in vitro transcription was performed using a Ribo™ RNAmax-T7 Kit (RiboBio) according to the manufacturer's instructions. All microinjections were performed using an Eclipse® Ti-U Eppendorf micromanipulation system (Nikon instrument). GV oocytes were harvested in an M2 medium with 1.5 μM milrinone to inhibit spontaneous GVBD. Reagents were microinjected at the following concentrations: *miR-425-5p* mimics at 100 nM, *miR-425-5p* inhibitors at 1 µM, *Crebzf* siRNA at 20 µM, and *Crebzf* cRNA at 500 ng/μL.

### Luciferase reporter assay

The potential binding sites of *miR-425-5p* in the *Crebzf* 3’ untranslated region (UTR) were predicted by TargetScan7.1. The wild-type 3’ UTR sequence or the mutated sequences of *Crebzf* were inserted into the XhoI-NotI site downstream of the pmiR-RB-Report™ vector (RiboBio). 293 T cells were cultured in 24-well plates in dulbecco's modified eagle medium (DMEM, Thermo Fisher Scientific) supplemented with 10% fetal bovine serum (FBS, Thermo Fisher Scientific) at 37 °C and then cotransfected with wild-type or a mutant construct (200 ng/well) and *miR-425-5p* mimics/inhibitors or the NC using Lipofectamine™ 2000 transfection reagent (Thermo Fisher Scientific). Twenty-four hours post transfection, cell lysates were analyzed for luciferase activity using a Duo-Lite Luciferase Assay System (Vazyme) according to the manufacturer's protocol.

### Statistical analysis

The data are presented as the mean ± standard deviation, unless otherwise stated. Statistical comparisons were made with Student's t test and analysis of variance (ANOVA) when appropriate using Prism 5 software (GraphPad, San Diego, CA, United States). When equal variances could not be assumed, Welch's test was used. *P* < 0.05 was considered to be significant. Each experiment was repeated at least three times.

## Results

### Result 1 *MiR-425-5p* expression increases in response to ROS accumulation during oocytes aging

**Fig. 1:**
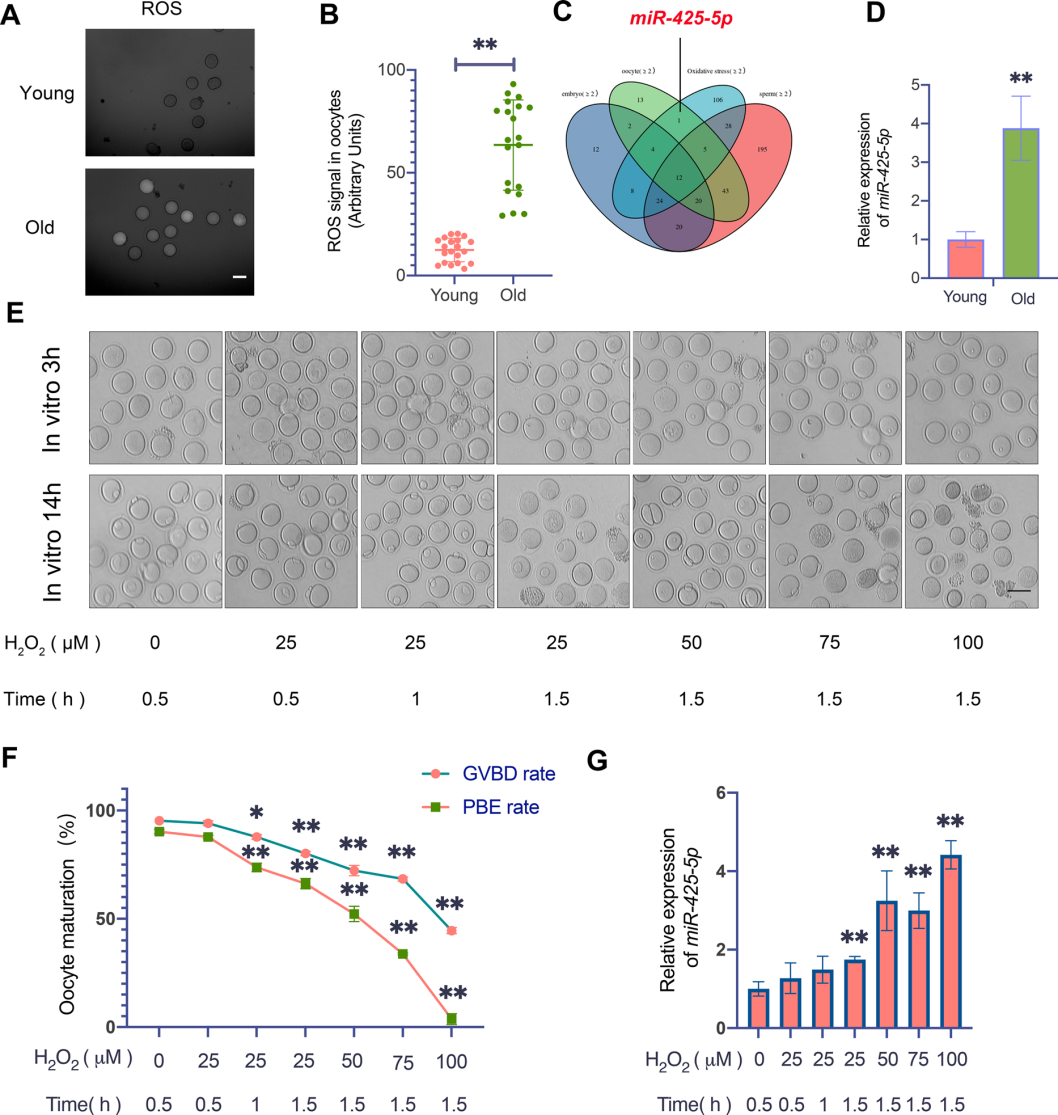
*MiR-425-5p* is upregulated in oocytes after in vitro oxidative stress induction and natural aging.** (A)** Upper and lower images: Representative images of small antral GV oocytes from young and reproductively older mice stained with CM-H2DCFAD fluorescence to evaluate ROS levels. Scale bar: 100 μm. **(B)** Quantitative analysis of the CM-H2DCFAD fluorescence intensity in GV oocytes from young and reproductively older mice (n = 20 in each group). *P* values were determined by the unpaired samples T-test. **(C)** Venn diagram for upregulated miRNAs in GV oocytes(green), embryos(cornflower blue), and sperm(pink) in reproductively older mice versus young mice. The miRNAs found to be exclusively upregulated in the GV oocytes were filtered with a publicly available miRNA expression database that included miRNAs altered after H_2_O_2_ exposure(sky blue). **(D)** *MiR-425-5p* expression levels in GV oocytes of young and reproductively older mice. *P* values were determined by the unpaired samples T-test. **(E)** Upper and lower images: Representative images of oocytes cultured in vitro for 3 h and 14 h after being treated with different concentrations of H_2_O_2_ for different times (0 μM*0.5 h, 25 μM*0.5 h, 25 μM*1 h, 25 μM*1.5 h, 50 μM*1.5 h, 75 μM*1.5 h and 100 μM*1.5 h). Scale bar = 100 μm. **(F)** The GVBD rate and PBE rate of oocytes after being treated with different concentrations of H_2_O_2_ for different times (n = 80 to 90 in each group). *P* values were determined by two-way ANOVA. **(G)** *MiR-425-5p* expression levels in GV oocytes after being treated with different concentrations of H_2_O_2_ for different times. *P* values were determined by one-way ANOVA. Results were representative of at least three independent experiments.*P < 0.05 and **P < 0.01

The deterioration of oocyte quality is a critical factor in the age-related decline of female fertility. Age-related oxidative damage has been identified as one of the key factors contributing to this decline in women and mice at advanced ages. Follicular fluid plays a crucial role in supporting oocyte development and maturation. To investigate the changes in OS levels with age, we allocated patients into two age groups and measured H_2_O_2_ levels in follicular fluid (Table 1). We found that older women had significantly higher H_2_O_2_ levels in their follicular fluid than younger women (Supplementary Fig. 1A). To further explore the effects of age on OS, we analyzed small antral GV oocytes from young and old mice and found that the levels of OS were significantly higher in the oocytes of older mice (Fig. 1A and B). Recently, miRNAs have been found to be profoundly involved in age-related processes [31]. To investigate the age-related alterations of miRNAs in mouse oocytes, we performed sequencing on GV oocytes from young and old mice. Additionally, we compared these results with those obtained from sperm and 8-cell embryos [32] (Supplementary Data 1). We found that 14 miRNAs were exclusively upregulated in the GV oocytes of older mice compared to younger mice, with a fold change greater than 2 (Fig. 1C). To determine whether the elevation of these miRNAs was a result of long-term ROS accumulation, we filtered the 14 miRNAs with a publicly available miRNA expression database that included miRNAs altered after H_2_O_2_ exposure [33]. We found that *miR-425-5p* was the only miRNA that was elevated under both natural aging and in vitro OS induction, with a highly conserved sequence among different species (Fig. 1C and Supplementary Fig. 1B). We further confirmed the increase of *miR-425-5p* levels in the oocytes of older mice compared to younger mice by RT-qPCR (Fig. 1D).

**Table 1:** Basic clinical characteristics of patients

Variables	Young	Advanced Age	P
Maternal age (years)	30.20 ± 2.96	39.00 ± 1.00	< 0.000001**
BMI(kg/m^2^)	21.51 ± 1.39	21.94 ± 1.36	0.328981
Duration of infertility(years)	3.00 ± 2.15	5.55 ± 5.14	0.047638*
Antral follicle count	15.65 ± 7.36	16.30 ± 8.13	0.792390
Basal FSH(IU/L)	9.81 ± 5.33	12.00 ± 9.69	0.381404
Basal LH(IU/L)	4.24 ± 2.66	3.46 ± 2.21	0.319512
Basal E2(pmol/L)	834.00 ± 1604.60	1913.80 ± 3535.88	0.221246
Basal PRL(mIU/L)	284.29 ± 119.18	257.10 ± 156.85	0.540737
Basal P (nmol/L)	3.06 ± 2.99	2.05 ± 1.73	0.198879
Basal T (nmol/L)	1.76 ± 0.55	1.21 ± 0.60	0.004478**
Total gonadotropin dose(IU)	2648.13 ± 940.95	2877.50 ± 854.80	0.424744
Duration of stimulation	11.10 ± 1.41	10.65 ± 1.52	0.337854
Number of oocytes retrieved	17.90 ± 6.55	12.25 ± 5.03	0.004051**
Mature oocytes rate(%)	85.99 ± 10.35	77.73 ± 13.62	0.037194*
Fertilization rate(%)	81.66 ± 15.81	86.95 ± 10.03	0.214090
Embryo cleavage rate(%)	99.64 ± 1.56	96.94 ± 6.61	0.083424
H_2_O_2_ concentration(nmol ml^−1^)	2.75 ± 0.74	5.28 ± 0.75	< 0.000100**

BMI, body mass index; FSH, follicle stimulating hormone; LH, luteinizing hormone; E2, estradiol; PRL, prolactin; P, progesterone; T, testosterone; H_2_O_2_, Hydrogen peroxide; Mature oocytes rate: number of MII oocytes / number of retrieved oocytes; Fertilization rate: number of fertilized oocytes / number of injected oocytes; Embryo Cleavage rate: number of cleaved embryo / number of fertilized oocytes. *P* values were determined by multiple T tests. *P < 0.05 and **P < 0.01 mean statistically significant

To investigate the relationship between OS and *miR-425-5p* in oocytes, we used an in vitro OS model by treating young immature GV oocytes with various concentrations and exposure times of H_2_O_2_ [34]. Our results showed that treating GV oocytes with 50 µM H_2_O_2_ for 90 min significantly decreased the maturation rate and increased the levels of *miR-425-5p* in oocytes, which corresponded to the results found in the oocytes of older mice (Fig. 1F and G). These findings suggest that *miR-425-5p* may respond to ROS accumulation and contribute to the compromised phenotype of oocytes during aging.

### Result 2 *MiR-425-5p* inhibition promotes oocyte maturation in reproductively old mice

**Fig. 2:**
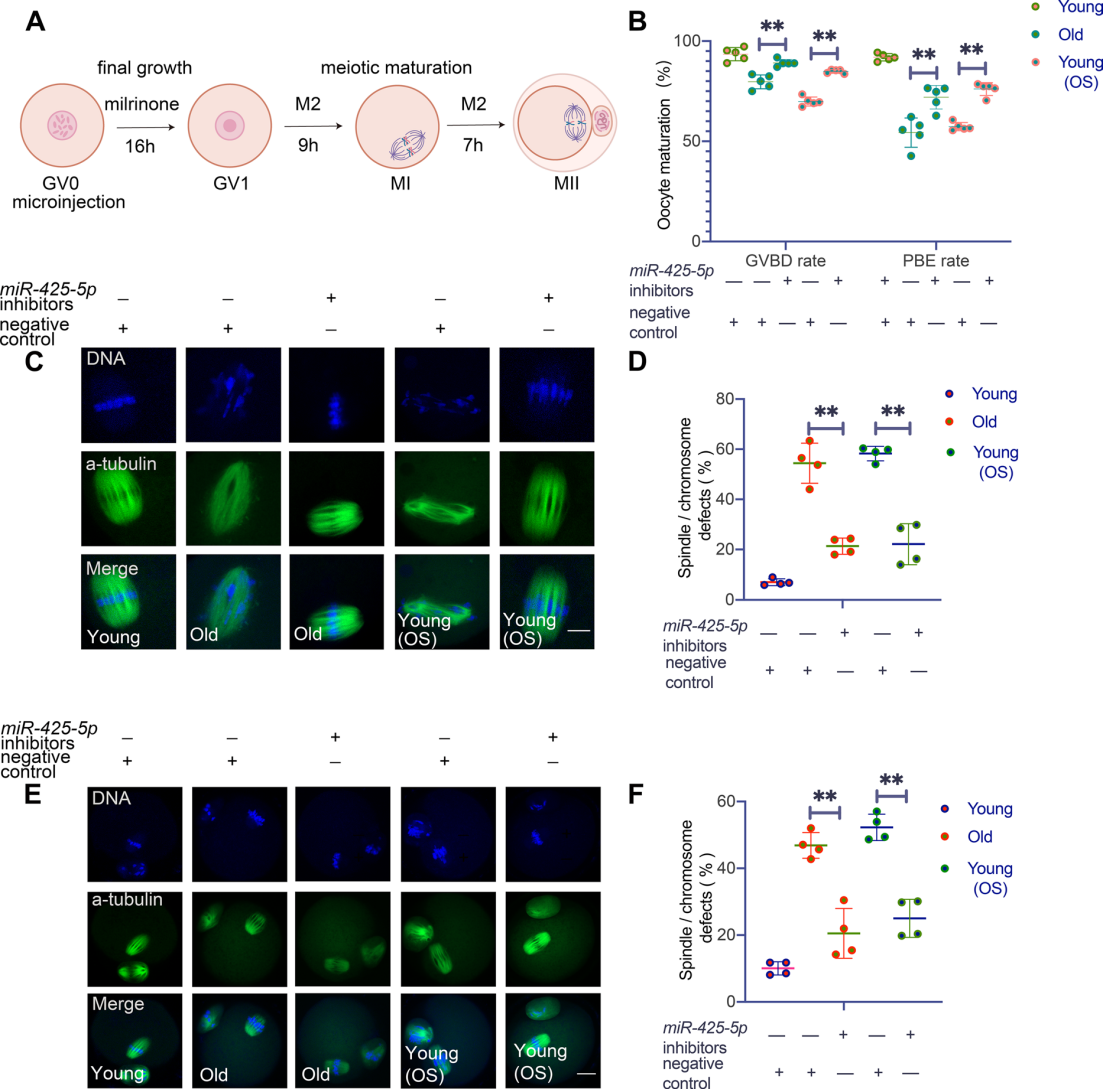
*MiR-425-5p* inhibition promotes oocyte maturation in reproductively old mice**. (A)** Experimental scheme of microinjection, final growth and meiotic maturation of oocytes. **(B)** The GVBD rate and PBE rate after microinjecting Young, Old, Young(OS) oocytes with *miR-425-5p* inhibitors or negative control (NC) (n = 90 to 100 in each group). *P* values were determined by two-way ANOVA. Representative images of spindle assembly and chromosome alignment at the Metaphase I (MI) **(C)** and Metaphase II (MII) **(E)** stages of Young, Old, Young(OS) oocytes after microinjecting with *miR-425-5p* inhibitors or NC. Scale bar: 10 μm in **(C)** and 20 μm in **(E)**. The proportion of oocytes with abnormal spindle and chromosome morphology at the MI **(D)** and MII **(F)** stages of Young, Old, Young(OS) oocytes after microinjecting with *miR-425-5p* inhibitors or NC (n = 50 to 60 in each group). *P* values were determined by one-way ANOVA. Results were representative of at least three independent experiments. *P < 0.05 and **P < 0.01

Based on the results presented above, we next investigated whether inhibiting *miR-425-5p* could mitigate the negative effects of natural aging and in vitro OS treatment on oocyte development. As depicted in Fig. 2A, GV oocytes were collected from young and reproductively older mice with or without in vitro OS induction, and were then treated with either *miR-425-5p* inhibitors or NC before further culture. The results showed that the maturation rates of the oocytes significantly improved after the application of *miR-425-5p* inhibitors in both reproductively old mice (GVBD from 79.70 ± 3.47% to 89.14 ± 1.71%; PBE from 54.35 ± 7.30% to 72.00 ± 5.89%) and in the in vitro OS model (GVBD from 69.88 ± 2.27% to 84.91 ± 0.97%; PBE from 57.33 ± 2.04% to 76.08 ± 3.24%) (Supplementary Fig. 2 and Fig. 2B).

Spindle assembly and chromosome segregation must be correctly commanded to guarantee the normal progress of oocyte meiosis. Given that *miR-425-5p* inhibition could improve oocyte maturation in reproductively old mice, we explored whether *miR-425-5p* inhibition could affect the chromosome segregation process. Oocytes at MI and MII stages were immunostained to observe the spindle structures and chromosome alignment. Our results showed that in contrast to the 'defective' structures observed in oocytes from reproductively older mice and those subjected to in vitro OS induction, which were characterized by the loss of regular microtubule poles and chromosomes displaying scattered, decondensed or disorganized patterns, a significantly higher percentage of 'normal' MI and MII oocytes, in which microtubules formed two opposite poles with orderly distributed chromosomes, were observed after the microinjection of *miR-425-5p* inhibitors (Fig. 2C to F). These findings indicate that compromised oocyte developmental competence in reproductively older females is likely due to ROS accumulation, and that lowering *miR-425-5p* has a beneficial impact on improving oocyte maturation in reproductively old females.

### Result 3 *Crebzf* downregulation correlates with increased *miR-425-5p* levels in aging oocytes

**Fig. 3:**
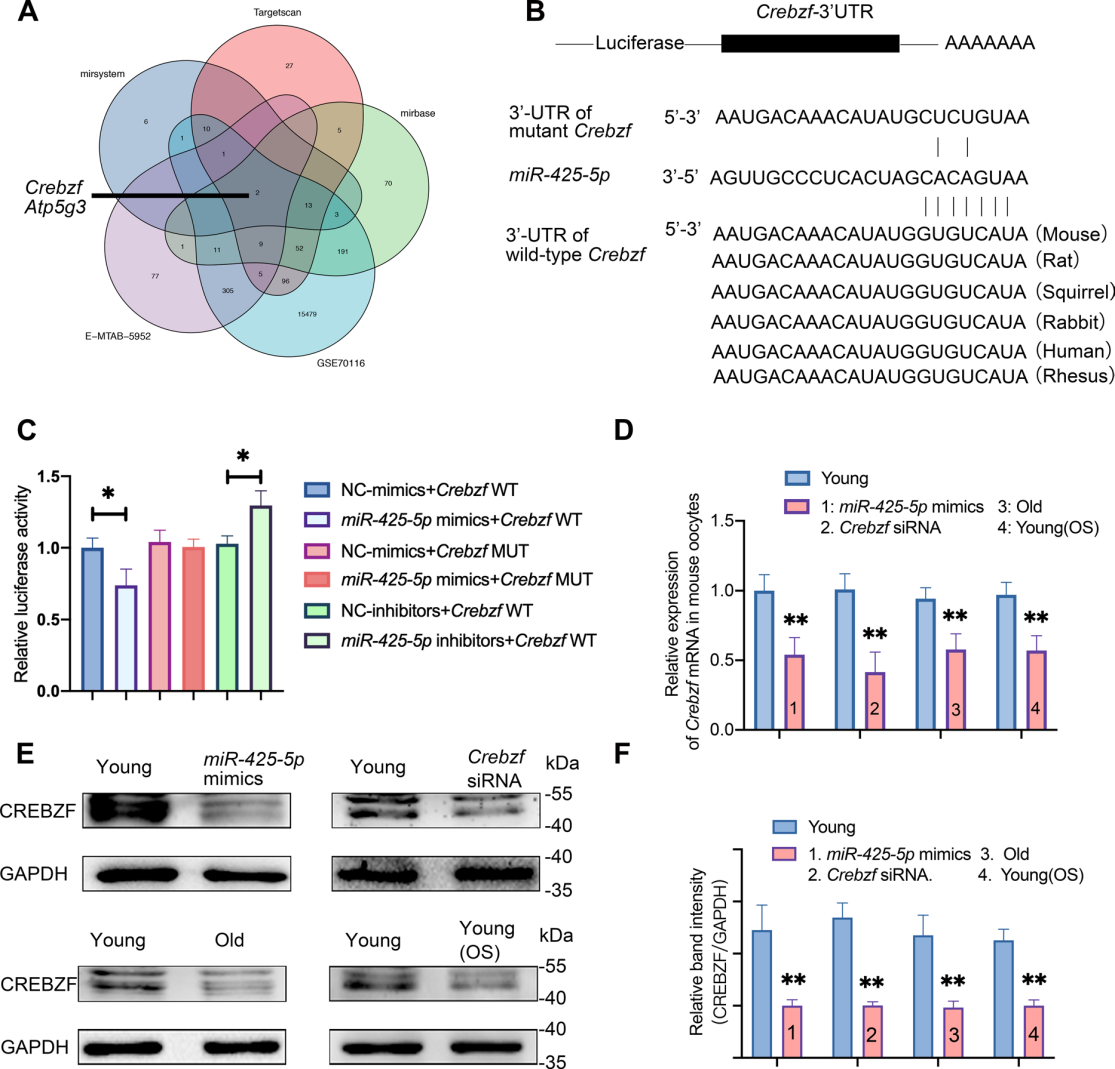
*Crebzf* is deregulated in oocytes as *miR-425-5p* levels rise with aging*. (A)* Venn diagram of *miR-425-5p*’s targets predicted by TargetScan (pink), miRbase (green) and miRSystems (cornflower blue); the data were further filtered using a publicly available database including genes upregulated in follicles from reproductively older mice (purple) and genes expressed at different developmental stages of oocytes (sky blue). **(B)** Sequence alignment of the *miR-425-5p* binding site with the wild-type (WT) or mutant (MUT) *Crebzf* 3’ UTR. **(C)** *MiR-425-5p* mimics, negative control (NC) mimics, *miR-425-5p* inhibitors and NC inhibitors were cotransfected with luciferase reporter constructs containing WT or MUT *Crebzf* 3’ UTR in 293 T cells, and relative luciferase activity was measured. *P* values were determined by one-way ANOVA. **(D)** Expression of *Crebzf* mRNA in Young GV1 oocytes compared with that in *Crebzf* siRNA, *miR-425-5p* mimics, Old and Young(OS) GV1 oocytes. *P* values were determined by multiple T tests. **(E)** Western blot comparing CREBZF in Young GV1 oocytes with that in *Crebzf* siRNA, *miR-425-5p* mimics, Old and Young(OS) GV1 oocytes. GAPDH was the internal loading control. **(F)** Relative band intensity of CREBZF after normalization to GAPDH**.** *P* values were determined by multiple T tests. Results were representative of at least three independent experiments.*P < 0.05 and **P < 0.01

To investigate the impact of *miR-425-5p* on oocyte aging, we employed databases such as TargetScan, miRBase, and miRSystems to predict miRNA targets. We then utilized a publicly available database that contained genes downregulated in reproductively old mouse follicles and genes expressed at various stages of oocyte development to filter the candidates (Fig. 3A and Supplementary Data 2) [11, 12]. After screening, we selected *Crebzf* and *Atp5g3* as strong candidates that may connect *miR-425-5p* with oocyte aging (Fig. 3A). It has been established that ATP production suppression after exposure to cellular stressors during oocyte development is associated with the aging process [11, 12], while little is known about the involvement of *Crebzf* in oocyte maturation [35]. We identified a predicted conserved target site for *miR-425-5p* in the 3’ UTR of *Crebzf* mRNA using the miRNA target prediction database TargetScan. To confirm whether *miR-425-5p* targets *Crebzf* through the predicted miRNA-binding sites, we cloned various *Crebzf* 3’ UTR regions containing wild-type and mutant *miR-425-5p* binding sites into luciferase reporters (Fig. 3B). Our results showed that *miR-425-5p* overexpression led to a substantial reduction in the expression of the wild-type reporter, but not the mutant reporter, in 293 T cells (Fig. 3C), confirming the binding of *miR-425-5p* to the 3’ UTR of *Crebzf*. We further investigated whether *miR-425-5p* could regulate the expression of *Crebzf* in mouse oocytes by performing RT‒qPCR and Western blotting. We observed a downward trend in both *Crebzf* mRNA and CREBZF protein expression upon *miR-425-5p* overexpression, indicating negative regulation of *Crebzf* by *miR-425-5p* (Fig. 3D to F). As *miR-425-5p* expression increased in oocytes of reproductively old females, we next explored the expression pattern of *Crebzf* in young and reproductively older individuals. As we expected, a decline in *Crebzf* expression at both mRNA and protein levels was observed in mouse oocytes with age. This effect was also observed after in vitro OS stimulation (Fig. 3D to F). Overall, these findings highlight *Crebzf* as a robust candidate that may mediate the age-related functions of *miR-425-5p* in mouse oocytes.

### Result 4In vivo inhibition of *miR-425-5p* ameliorates oocytequality by restoring* Crebzf *levels in reproductively old mice

**Fig. 4:**
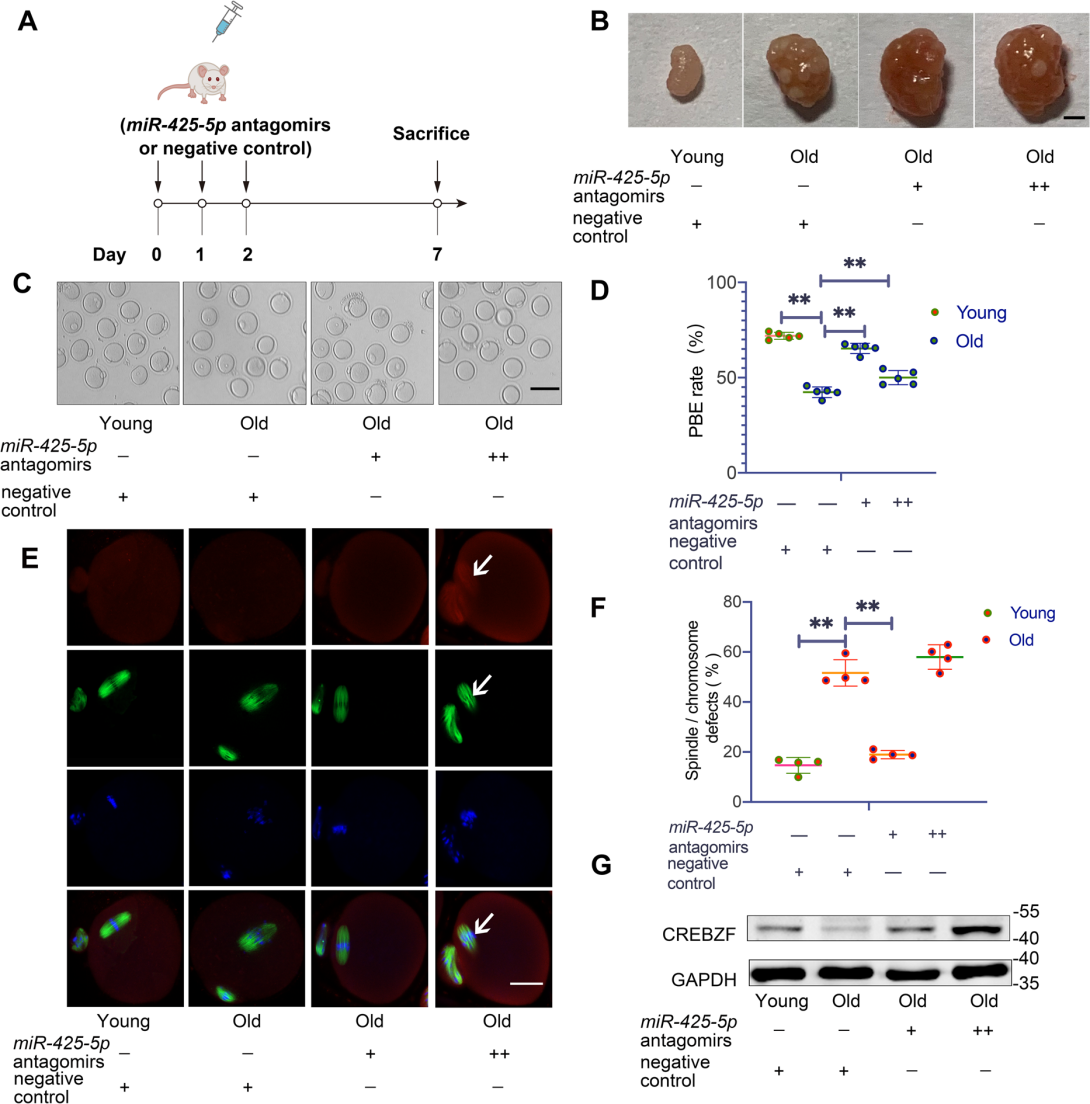
In vivo inhibition of *miR-425-5p* ameliorates oocyte quality by restoring *Crebzf* levels in reproductively old mice.** (A)** Schematic diagram of the in vivo experiments. Young female mice were injected with negative control(Young NC). Reproductively old female mice received injections of either *miR-425-5p* antagomirs at a medium dose of 50 mg/kg (Old +) or a high dose of 80 mg/kg (Old + +), or NC (Old NC) (n = 3 in each group). **(B)** Representative images of ovaries in Young (NC), Old (NC), Old ( +) and Old (+ +) groups. Scale bar: 1 mm. **(C)** Representative images of oocytes cultured in vitro for 14 h. Scale bar: 100 μm. **(D)** The PBE rates of oocytes in different groups (n = 30 to 35 in each group). *P* values were determined by one-way ANOVA. **(E)** Representative images of spindle assembly and chromosome alignment in different groups. Arrows indicate colocalization of CREBZF and defected spindle. Scale bar: 20 μm. **(F)** The proportion of oocytes with abnormal spindle and chromosome morphology at the MII stages (n = 30 to 35 in each group)**. (G)** Western blot comparing CREBZF levels in mouse ovaries. GAPDH was the internal loading controls. Results were representative of at least three independent experiments. *P < 0.05 and **P < 0.01

Based on the results of our previous experiments, we designed and conducted in vivo experiments to investigate the impact of in vivo *miR-425-5p* silencing on oocyte maturation in reproductively old mice (Fig. 4A). We extracted the ovaries and weighed them, finding that the relative ovarian weight did not differ significantly among the medium dose, high dose, and old control groups (Fig. 4B and Supplementary Fig. 3A). Subsequently, we harvested GV oocytes and cultured them in vitro for further analysis. Compared to the control group, the rates of oocyte maturation showed significant and slight increases in the medium and high dose groups, respectively (Fig. 4C and D). Notably, oocytes in the medium dose group had fewer meiotic errors, while oocytes in the high dose group had more meiotic errors than those in the old control group (Fig. 4E and F). We observed the colocalization of CREBZF and the defected spindles in the high dose group, unlike the barely absent or extremely low expression level of CREBZF in MII oocytes of young or old controls (Fig. 4E). In this context, we inferred that the disruption of the meiotic process in the high dose group might be due to *Crebzf* overexpression. Based on the above results, we explored whether and how *Crebzf* levels changed after in vivo silencing of *miR-425-5p*. RT-qPCR results from oocytes and Western Blot results from ovaries indicated the increase of *Crebzf* in the *miR-425-5p* antagomirs groups. *Crebzf* levels in the medium dose group increased similarly to those of young controls, while they increased excessively in the high-dose group compared to young controls (Fig. 4G and Supplementary Fig. 3B). These findings emphasized the necessity of maintaining adequate *Crebzf* levels in oocyte maturation and indicated that in vivo treatment with a medium dose of *miR-425-5p* antagomirs might improve impaired oocyte development in reproductively old females.

### Result 5 Overexpression of *miR-425-5p* hinders oocyte maturation by targeting *Crebzf*

**Fig. 5:**
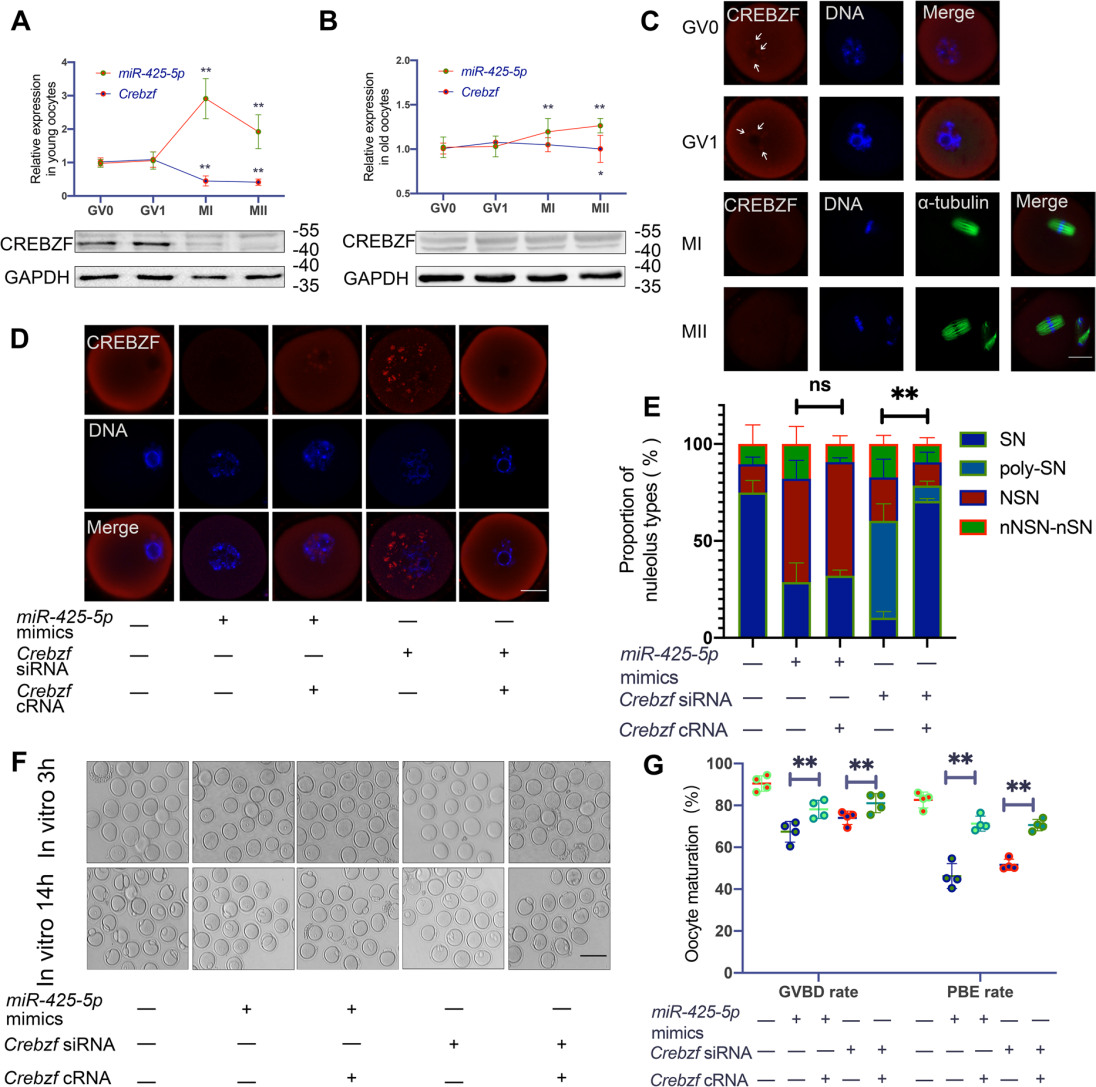
*MiR-425-5p* overexpression interferes with oocyte maturation by targeting *Crebzf.* Upper: Relative expression of *miR-425-5p* and *Crebzf* mRNA at different developmental stages of Young oocytes.* P* values were determined by two-way ANOVA. Lower: Western blot comparing CREBZF at different developmental stages of Young oocytes. GAPDH was the internal loading control. **(B)** Upper: Relative expression of *miR-425-5p* and *Crebzf* mRNA at different developmental stages of Old oocytes.* P* values were determined by two-way ANOVA. Lower: Western blot comparing CREBZF at different developmental stages of Old oocytes. GAPDH was the internal loading control. **(C)** Representative images of CREBZF localization at different developmental stages of Young oocytes. Arrows indicate localization of CREBZF around the nucleolus. Scale bar: 20 μm. **(D)** Representative images of chromatin configurations in Young GV1 oocytes after microinjecting with *miR-425-5p* mimics, *miR-425-5p* mimics + *Crebzf* cRNA, *Crebzf* siRNA or *Crebzf* siRNA + *Crebzf* cRNA. Scale bar: 20 μm. **(E)** Proportion of different chromatin configurations in Young GV1 oocytes after microinjecting with *miR-425-5p* mimics, *miR-425-5p* mimics + *Crebzf* cRNA, *Crebzf* siRNA or *Crebzf* siRNA + *Crebzf* cRNA(n = 60 in each group). **(F)** Representative images of oocytes cultured in vitro for 3 and 14 h after microinjecting with *miR-425-5p* mimics, *miR-425-5p* mimics + *Crebzf* cRNA, *Crebzf* siRNA or *Crebzf* siRNA + *Crebzf* cRNA. Scale bar = 100 μm. **(G)** The GVBD rate and PBE rate after microinjecting with *miR-425-5p* mimics, *miR-425-5p* mimics + *Crebzf* cRNA, *Crebzf* siRNA or *Crebzf* siRNA + *Crebzf* cRNA (n = 70 to 80 in each group). *P* values were determined by two-way ANOVA. Results were representative of at least three independent experiments. *P < 0.05 and **P < 0.01

To explore the possible mechanism by which *miR-425-5p* regulation of *Crebzf* affects oocyte quality, we detected the expression pattern of *miR-425-5p* and *Crebzf* at different developmental stages of the oocytes in young and reproductively old mice. RT-qPCR and Western blotting results revealed higher expression of *miR-425-5p* in young oocytes following meiotic resumption, whereas *Crebzf* showed an opposite expression pattern (Fig. 5A). Intriguingly, this expressional pattern was not noticeable in old oocytes, in which *Crebzf* was nearly undetectable during the GV0 to MII transition (Fig. 5B). To investigate how *Crebzf* is involved in oocyte maturation, we next observed the cellular localization of the CREBZF protein in young oocytes. We found its enrichment in the cytoplasm and around the nucleolus in GV0 and GV1 oocytes (Fig. 5C), which indicated a potential role for CREBZF in the chromatin modification process. NSN-to-SN transition is a crucial aspect of oocyte chromatin modification before gaining full developmental competence. Therefore, we investigated the chromatin structures of oocytes after microinjecting with *miR-425-5p* mimics or siRNA targeting *Crebzf* (Fig. 5D and E). Results showed that GV1 oocytes in NC group mostly present SN-type structures with thread-like, compacted nuclear chromatin with a ring around the nucleolus. While GV1 oocytes in *miR-425-5p* mimics group mainly exhibited non NSN and non SN structure (nNSN-nSN) which is characterized by diffuse nuclear chromatin without a ring around the nucleolus. Notably, *Crebzf* downregulation generated a configuration with a minor difference from the SN structure, distinguished by the presence of multiple nucleoli of varying sizes surrounding a nucleolus-like body of greater size (poly-SN) (Fig. 5D and E). Without observing the dramatic effects of *Crebzf* downregulation on NSN to SN transition, we investigated whether oocyte maturation was affected. Interestingly, upon overexpression of *miR-425-5p* or downregulation of *Crebzf*, the rates of GVBD and PBE both showed a consistent decreasing trend, which was more pronounced for the former (Fig. 5F and G). Following this, we observed the spindle assembly and chromosome alignment at the MI and MII stages. Results found that chromatin segregation was perturbed in both instances, with a more significant proportion of faulty spindles and irregular chromatin alignment for the former (Supplementary Fig. 4). Exogenous supplementation with *Crebzf* may partially or substantially offset these negative phenotypes induced by *miR-425-5p* overexpression or *Crebzf* downregulation, respectively (Fig. 5F,G and Supplementary Fig. 4). Taken together, our findings suggest that *miR-425-5p* targets *Crebzf* to disrupt oocyte maturation and that *Crebzf* downregulation owing to *miR-425-5p* overexpression may explain at least some of the oocyte maturation errors addressed in reproductively old females.

### Result 6 *MiR-425-5p* overexpression downregulates H3K4me3 by targeting *Crebzf* to impede transcriptional silencing in GV oocytes

**Fig. 6:**
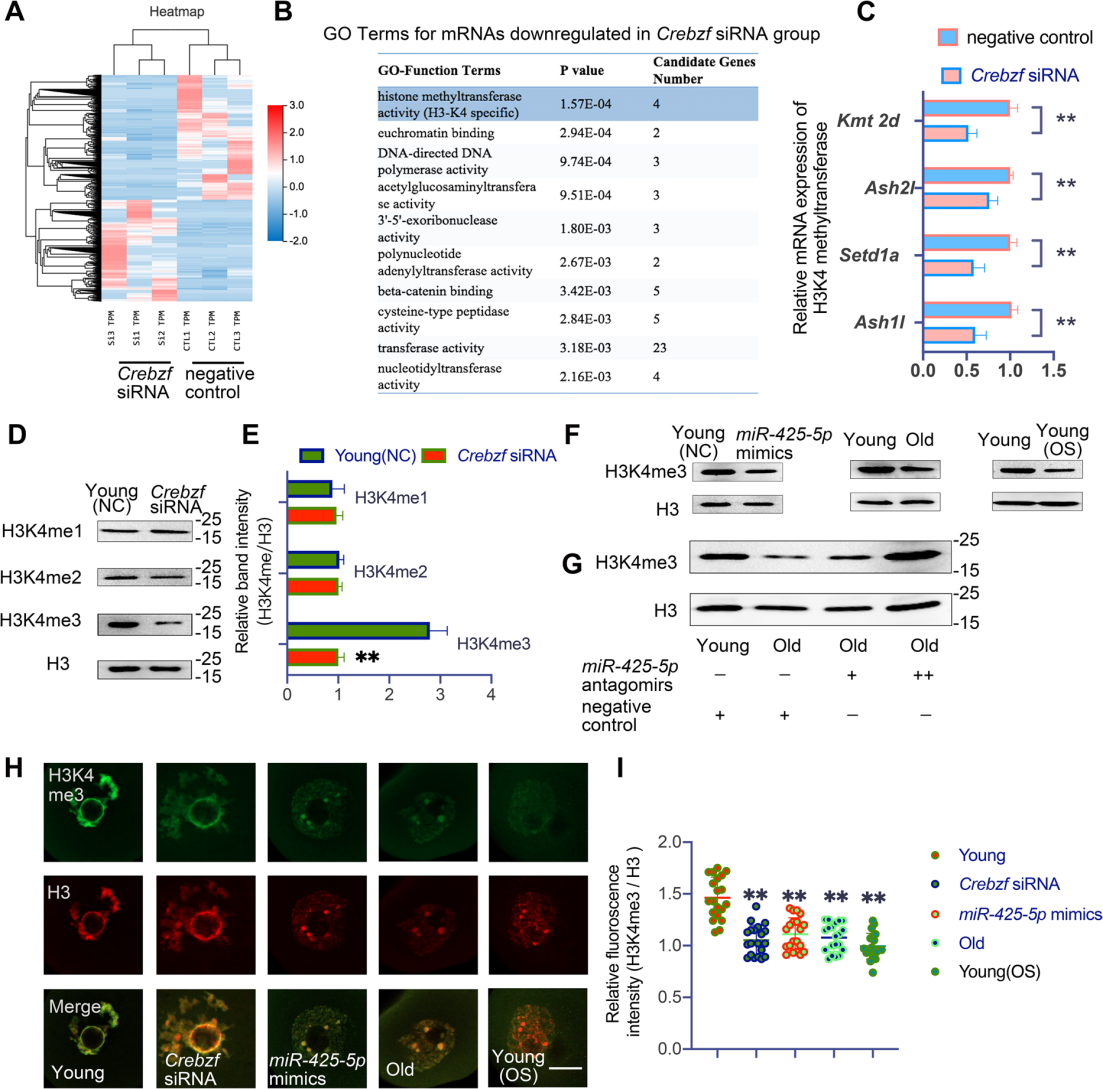
*MiR-425-5p* targets *Crebzf* to regulate H3K4 trimethylation in oocytes.** (A)** Heatmap of the significantly differentially expressed genes (adjusted *P* value < 0.05 and fold change > 2) in Young GV1 oocytes microinjected with negative control(NC) or *Crebzf* siRNA. The color indicates the expression level after z-scoring. **(B)** GO terms enriched with downregulated genes in oocytes microinjected with *Crebzf* siRNA versus NC. The ten most highly enriched categories were shown. **(C)** RT-qPCR validation of candidate genes in the most highly enriched category of RNA-Seq data.* P* values were determined by multiple T tests. **(D)** Western blot comparing H3K4me1, H3K4me2 and H3K4me3 in NC and *Crebzf* siRNA GV1 oocytes. H3 was the internal loading control. **(E)** Relative band intensity of H3K4me1, H3K4me2 and H3K4me3 after normalization to H3 in NC and *Crebzf* siRNA GV1 oocytes. *P* values were determined by multiple T tests. **(F)** Western blot comparing H3K4me3 in NC, *miR-425-5p* mimics and Young(OS) GV1 oocytes. H3 was the internal loading control. **(G)** Western blot comparing H3K4me3 in ovaries from Young (NC), Old (NC), Old ( +) and Old (+ +) groups. H3 was the internal loading control. **(H)** Representative images of H3K4me3 localization in Young(NC), *Crebzf* siRNA, *miR-425-5p* mimics, Old and Young(OS) GV1 oocytes. Scale bar: 10 μm. **(I)** Quantification of the immunofluorescence intensity of H3K4me3 after normalization to H3(n = 30 to 40 in each group). *P* values were determined by one-way ANOVA. Results were representative of at least three independent experiments. *P < 0.05 and **P < 0.01

In order to investigate how *Crebzf* affects oocyte maturation through chromatin modification events independent of structural changes, we conducted single-cell RNA-seq analyses on control oocytes and *Crebzf*-deficient oocytes that were microinjected with *Crebzf* siRNA. As shown by hierarchical clustering (Fig. 6A), we examined 220,033 mRNAs with detectable expression in a single oocyte and found that 101 transcripts had higher levels and 150 transcripts had lower levels in *Crebzf*-deficient oocytes compared to controls (log2-fold change > 1; adjusted *P* value (Q) < 0.05). Upon performing the Gene Ontology (GO) function analysis, we found that H3K4 methylation was the most significantly enriched category among downregulated mRNAs (Fig. 6B). We confirmed the decrease of four histone methyltransferase mRNAs in *Crebzf*-deficient oocytes through qRT‒PCR (Fig. 6C).

**Fig. 7:**
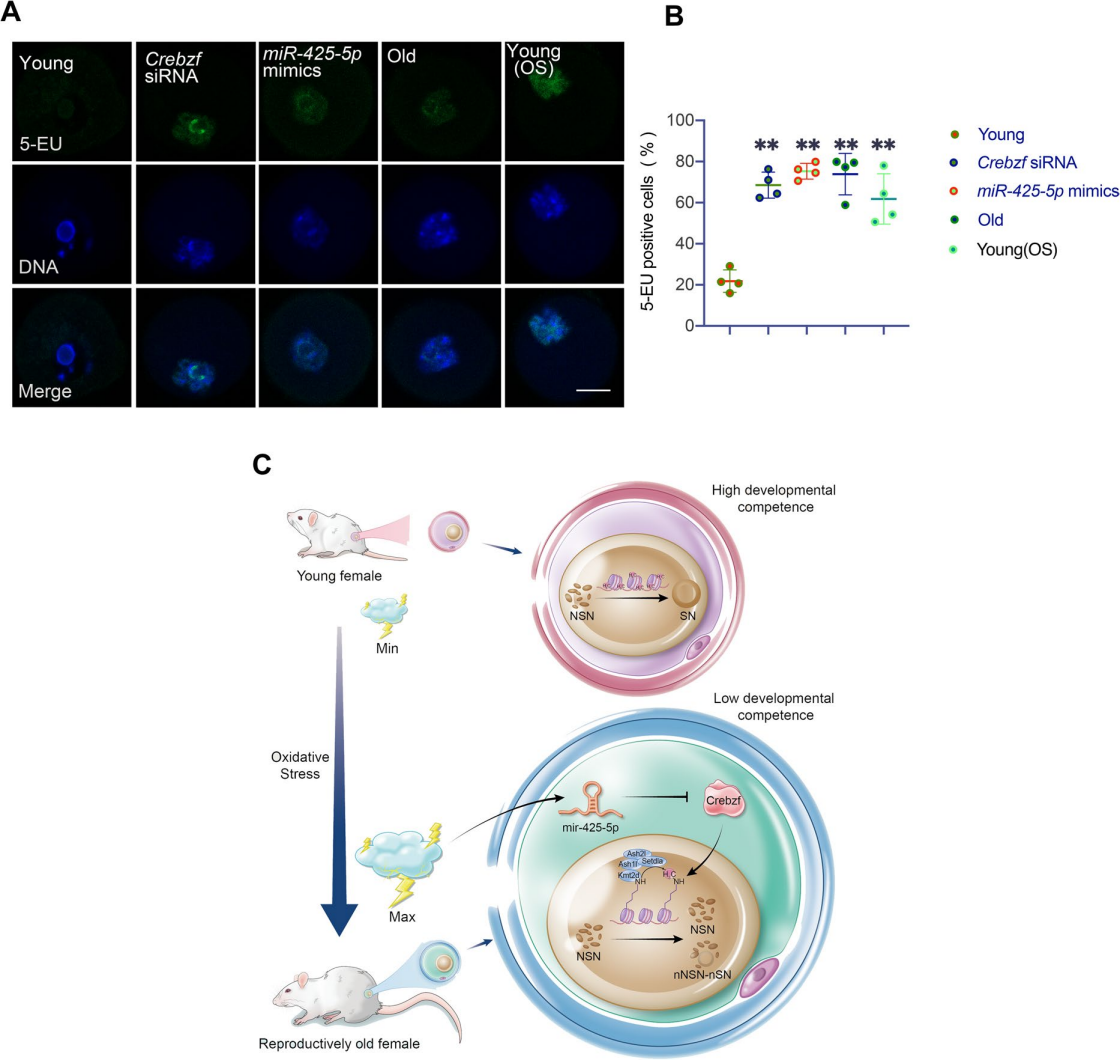
*MiR-425-5p* overexpression targets *Crebzf* to impede transcriptional silencing by deregulating H3K4me3 in GV oocytes.** (A)** Representative images of immunofluorescence analysis on Young, *Crebzf* siRNA, *miR-425-5p* mimics, Old and Young(OS) GV1 oocytes after culturing with 5-ethynyl uridine (EU). **(B)** Proportion of the 5-EU-positive oocytes in Young(NC), *Crebzf* siRNA, *miR-425-5p* mimics, Old and Young(OS) GV1 oocytes(n = 30 to 40 in each group). *P* values were determined by one-way ANOVA. Scale bar: 20 μm. **(C)** Working model of *miR-425-5p*. Age-related accumulation of ROS induces *miR-425-5p-Crebzf* signaling, reducing oocyte developmental competence by deregulating a group of histone methyltransferase mRNAs. Results were representative of at least three independent experiments. *P < 0.05 and **P < 0.01

H3K4 methylation proceeds in a graded pattern, producing mono-, di-, or trimethylated H3K4 (H3K4me1, H3K4me2, and H3K4me3, respectively). Therefore, we explored the expression pattern of H3K4me1, H3K4me2, and H3K4me3 in *Crebzf*-deficient and control oocytes. Results showed a dramatic decrease in H3K4me3 levels in *Crebzf*-deficient oocytes, while no change was observed in the H3K4me1 and H3K4me2 levels (Fig. 6D and E). *MiR-425-5p* overexpression, in vitro OS stimulation, and natural aging also led to the decrease of H3K4me3 level in oocytes (Fig. 6F and Supplementary Fig. 5A). Furthermore, in vivo administration of *miR-425-5p* antagomirs could reverse the decreased H3K4me3 levels in the ovaries of reproductively old mice (Fig. 6G and Supplementary Fig. 5B). To examine H3K4me3 levels in individual oocytes, we conducted immunofluorescence analyses and observed significant variations in the levels of histone H3K4me3 in GV1 oocytes following *Crebzf* downregulation, *miR-425-5p* overexpression, in vitro OS stimulation and natural aging (Fig. 6H and I). Global transcriptional silencing and the acquisition of complete developmental competence are closely linked events during the final stages of oocyte growth. Recent literature has identified H3K4me3 as a marker of transcriptional silencing in oocytes [36]. Given that oocytes lacking *Crebzf* exhibited dysregulated expression of H3K4me3, we assumed *Crebzf* is essential for oocyte transcriptional silencing. Therefore, we used a commonly used molecular biology reagent, 5-EU, to examine transcription activity on a global scale. Consistent with our assumption, most GV1 oocytes lacking *Crebzf* failed to undergo transcriptional silencing, which was phenocopied by *miR-425-5p* overexpression, in vitro OS induction and natural aging (Fig. 7A and B). Based on the aforementioned findings, we concluded that *miR-425-5p*’s regulation of *Crebzf* hinders the transcriptional silencing process of oocytes by downregulating the H3K4me3 modification in reproductively old females.

## Discussion

Errors in oocyte chromosomal segregation during meiosis are a prevalent cause of numerical chromosome abnormalities in human embryos derived from mothers of advanced maternal age [37]. Despite overwhelming evidence pointing out that even subtle defects in the chromatin modification process before meiotic resumption directly impact chromosome segregation, the mechanism by which maternal age affects chromatin modification is not well understood. Chromatin modification is a dynamic process orchestrated by ATP-dependent changes in chromatin structure and other epigenetic modifications, including DNA methylation, posttranslational histone modification, or noncoding RNAs (ncRNAs) [38]. Here, we propose a new crosstalk whereby ncRNAs can modulate chromatin modification by changing chromatin structures and participating in posttranslational histone modification. Specifically, we identify the non-coding RNA *miR-425-5p* as an epigenetic regulator and chromatin remodeler that responds to ROS accumulation in the aging process (Fig. 1 and Supplementary Fig. 1). Overexpression of *miR-425-5p* effectively mimics compromised phenotypes observed in oocytes of reproductively old females by targeting *ATP5g3* and *Crebzf* (Fig. 3). ATP synthase has been well characterized in oocytes, and the inability to produce ATP is closely linked with developmental failure in old oocytes [39]. As a subunit of mitochondrial ATP synthase, *ATP5g3* was proven to decrease in an age-dependent manner [40, 41], which might partially account for oocyte maturation defects observed in reproductively old females. With little knowledge about *Crebzf*, whether and how it is involved in oocyte maturation remains an open question. Our results demonstrate that inhibition of *Crebzf* impaired the oocyte meiotic process as overexpression of *miR-425-5p*, and the oocyte meiotic defects caused by *miR-425-5p* overexpression were largely abolished by ectopic expression of *Crebzf*, indicating that *Crebzf* also mediates the age-related function of *miR-425-5p* in oocyte maturation (Fig. 5, Supplementary Fig. 4). Notably, either *Crebzf* loss or gain led to oocyte abnormalities, emphasizing the importance of *miR-425-5p* in buffering *Crebzf* levels. During the GV0 to MII transition of oocytes, *miR-425-5p* and *Crebzf* presented opposite expression signatures. *MiR-425-5p* showed higher abundance with increasing maturation, while *Crebzf* became less abundant through these stages (Fig. 5). Consequently, *miR-425-5p* is evident as a unique regulator of maternal mRNA decay, which is vital for transitioning from a maternal role to that of a zygotic function after fertilization. In a previous study, a comparison of human MII stage oocytes from young and older women revealed inconsistent expression of *miR-425-5p* [42]. In our study, we observed higher levels of *miR-425-5p* in the GV0 stage oocytes of older mice compared to young oocytes (Fig. 1D). While *miR-425-5p* levels showed a significant increase from the GV0 to MII stage in young oocytes, there was less pronounced change in its levels in aged oocytes (Fig. 5A), which might resulted in limited difference in *miR-425-5p* levels between young and aged oocytes at the MII stage.

Transcriptional activity is essentially shut down in the final stages of oocyte growth, and does not resume until the two-cell embryo stage in mice [43]. Histones, which make up the majority of chromatin, serve as important regulators of transcription [44]. Modifications to histones can result in either transcriptionally active or silent chromatin states. Acetylation, for example, leads to gene activation, while methylation can have varying effects depending on the specific methylation pattern [45]. Although both histone (K) and arginine residues (R) can be methylated, lysine residues on histone tails (H3 and H4) are more commonly observed to be methylated [46]. In the past decade, there has been a considerable focus on the broad genomic distribution patterns for H3K4me3. As H3K4me3 is preferentially enriched at the transcription start sites of active genes in somatic cells, the mark is less conspicuous at transcription start sites in oocytes [47]. It is currently accepted that H3K4me3 serves as a transcription silent marker in oocytes and may have a transcription-independent role in the meiotic process [48–50]. Recent studies have demonstrated that the age-related decrease in H3K4me3 expression in oocytes is consistent with the decline in pregnancy rates observed in assisted reproduction [51]. The regulation of H3K4me3 is tightly controlled by histone lysine demethylases (KDMs) and histone lysine methyltransferases (KMTs), with SET Domain Containing 1 (SETD1)-based histone methyltransferase playing a predominant role in most cell types [52, 53]. However, the SET domain of SETD1-based histone methyltransferase is susceptible to oxidation, making it vulnerable to the effects of OS, which can lead to lower H3K4me3 levels [54]. This effect may partially explain the reduced levels of H3K4me3 observed in oocytes collected from reproductively older mice (Fig. 6 and Supplementary Fig. 5).

*Crebzf* plays a critical role in bridging the gap between metabolism and cell growth by acting as a transcription cofactor through the formation of heterodimers with other proteins [55, 56]. Its identification initially came through its interaction with the herpes simplex virus-1 related cellular protein (HCF-1) [57], which is required for the recruitment of histone methyltransferases leading to H3K4me3 [58]. The efficient binding of CREBZF to HCF-1 suggests its possible involvement in the HCF-1-related chromatin modification process. This could either be through sequestering HCF-1 from other cellular factors or by acting as a mediator between HCF-1 and its associated factors.

A rapidly growing body of evidence has identified miRNAs as potential targets for curing disease [59], while their potential therapeutic use in the treatment of infertility was hardly mentioned. In our study, we conducted both in vitro and in vivo treatments targeting *miR-425-5p* on reproductively old mice (Fig. 2, 4 and Supplementary Fig. 5). Our findings indicate that these treatments have beneficial effects, which suggests that the use of drugs such as miRNA antagonists may hold promise for infertility treatment. However, as demonstrated in this study, it has been observed that an excessive amount of *miR-425-5p* antagomirs negatively impacts oocyte maturation. Consequently, it becomes crucial to carefully consider the potential risks and limitations associated with the utilization of *miR-425-5p* antagomirs in therapeutic approaches targeting ovarian aging. Therefore, it is imperative to conduct further well-designed studies before making any definitive claims or assertions regarding the potential use of *miR-425-5p* antagomirs in ovarian aging therapy. Additional research will help to provide a more comprehensive understanding of its effectiveness and safety in such applications.

Collectively, our findings strongly suggest a mechanism in which ROS accumulation due to aging induces *miR-425-5p*-*Crebzf* signaling. This mechanism inhibits a developmental switch in the oocyte by regulating a group of histone methyltransferase mRNAs, as shown in Fig. 7C. Furthermore, our research indicates a promising approach for improving the deterioration of oocyte quality caused by aging and OS by targeting *miR-425-5p*. By doing so, chromatin structure can be switched, global transcriptional silencing can be induced, and a developmentally competent oocyte can be produced.

### Supplementary Information

Below is the link to the electronic supplementary material.

Supplementary file1 (DOCX 2228 KB)

## Data Availability

The datasets supporting the conclusions of this article are available in the National Center for Biotechnology Information (NCBI) database (under BioProject accession number PRJNA744948) and included within the article and its additional files.
