# Supplementary material for: MiR-425-5p suppression of Crebzf regulates oocyte aging via chromatin modification
Source: GeroScience. 2023 Aug 3;46(4):3723–42. doi: 10.1007/s11357-023-00875-6 (PMC11226420; doi:10.1007/s11357-023-00875-6)
Supplement: Supplementary file 1 — Supplementary file1 (DOCX 2228 KB) [file 11357_2023_875_MOESM1_ESM.docx]

Supplementary Materials for

***MiR-425-5p* suppression of *Crebzf* regulates oocyte aging via chromatin modification**

Kadiliya Jueraitetibaike ^1^ †, Ting Tang ^1, 2^ †, Rujun Ma ^1^ †, Shanmeizi Zhao ^3^ †, Ronghua Wu ^1^, Jian Zhong ^2^, Yang Yang ^4^, Xuan Huang ^1^, Xi Cheng ^1^, Cheng Zhou ^1^, Hong Zhang ^1^, Lu Zheng ^1^, Xie Ge ^1^, Li Chen ^1^ and Bing Yao ^1, 2^ *

*Corresponding author. Bing Yao ([yaobing@nju.edu.cn](mailto:yaobing@nju.edu.cn))

**This PDF file includes:**

Figs. S1 to S5

Table S1


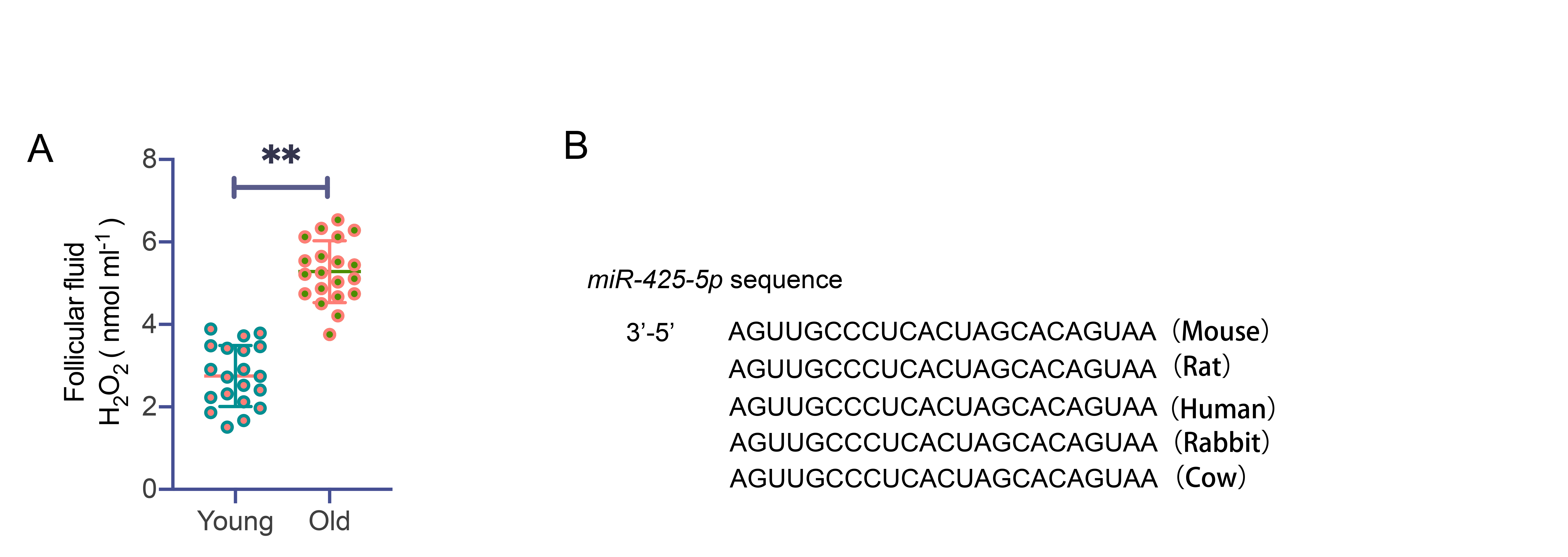


**Fig. S1. (A)** H_2_O_2_ concentration in follicular fluid collected from young and reproductively older women (n=20 in each group). *P* values were determined by the unpaired samples T-test. **(B)**Sequence of *miR-425-5p* in different species. **P* < 0.05 and ***P* < 0.01.

**
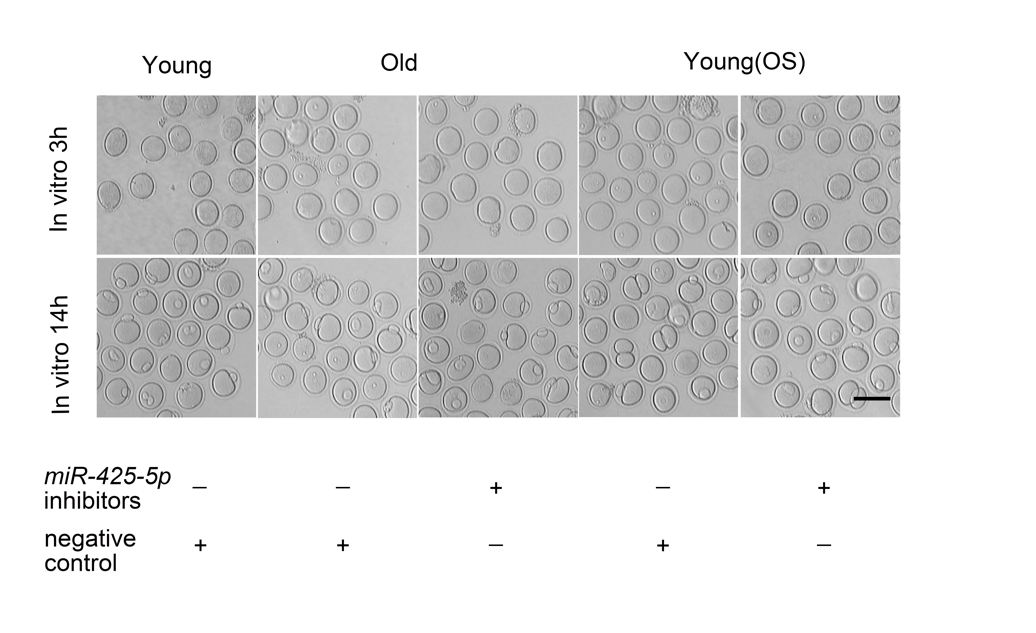
**

**Fig. S2.** Upper and lower images: Representative images of oocytes cultured in vitro for 3 hours and 14 hours after microinjecting Young, Old, Young(OS) oocytes with *miR-425-5p* inhibitors or negative control. Scale bar = 100 μm.


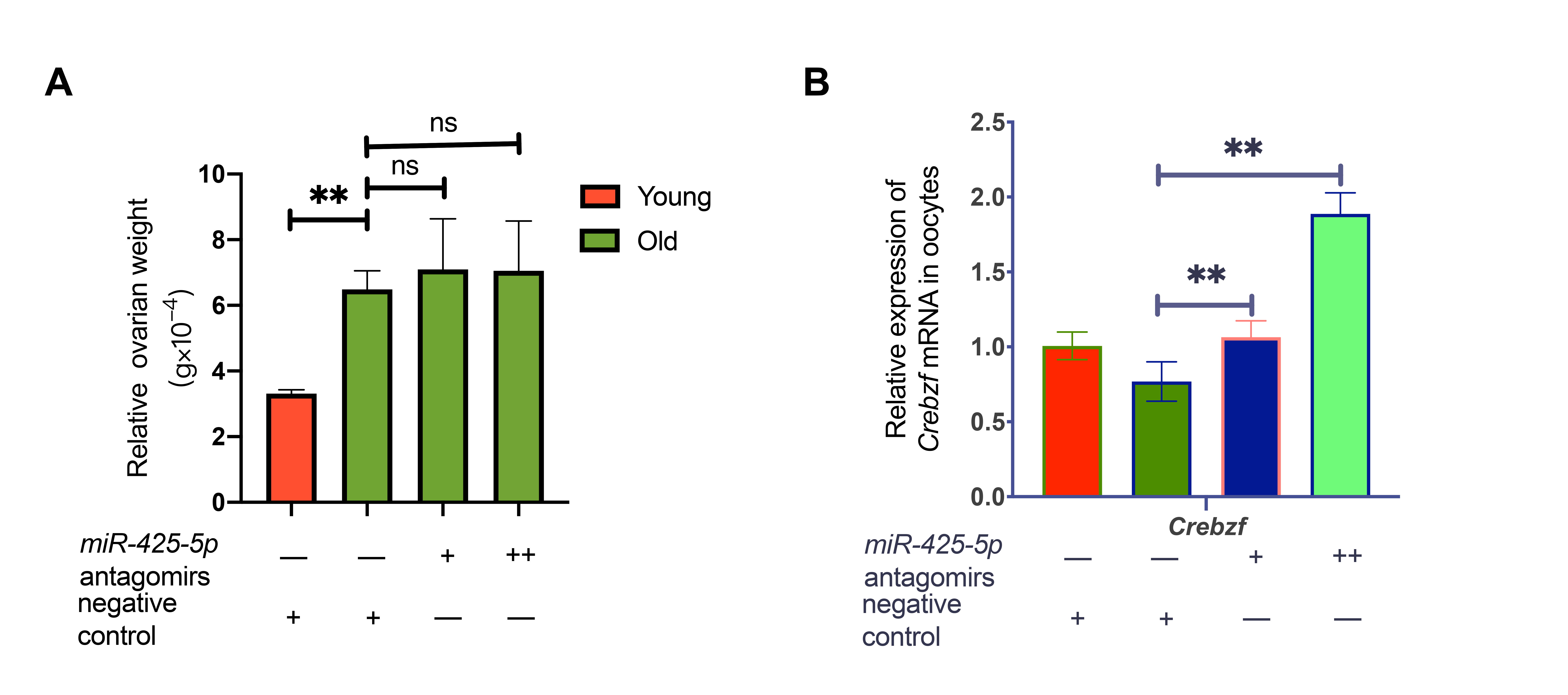


**Fig. S3. (A)** Relative ovarian weight in Young (NC), Old (NC), Old (+) and Old (++) groups. ns: not significant. *P* values were determined by one-way ANOVA. **(B)** RT-qPCR results of *Crebzf* mRNA levels in mouse oocytes. *P* values were determined by one-way ANOVA. Results were representative of at least three independent experiments. **P* < 0.05 and ***P* < 0.01.

**
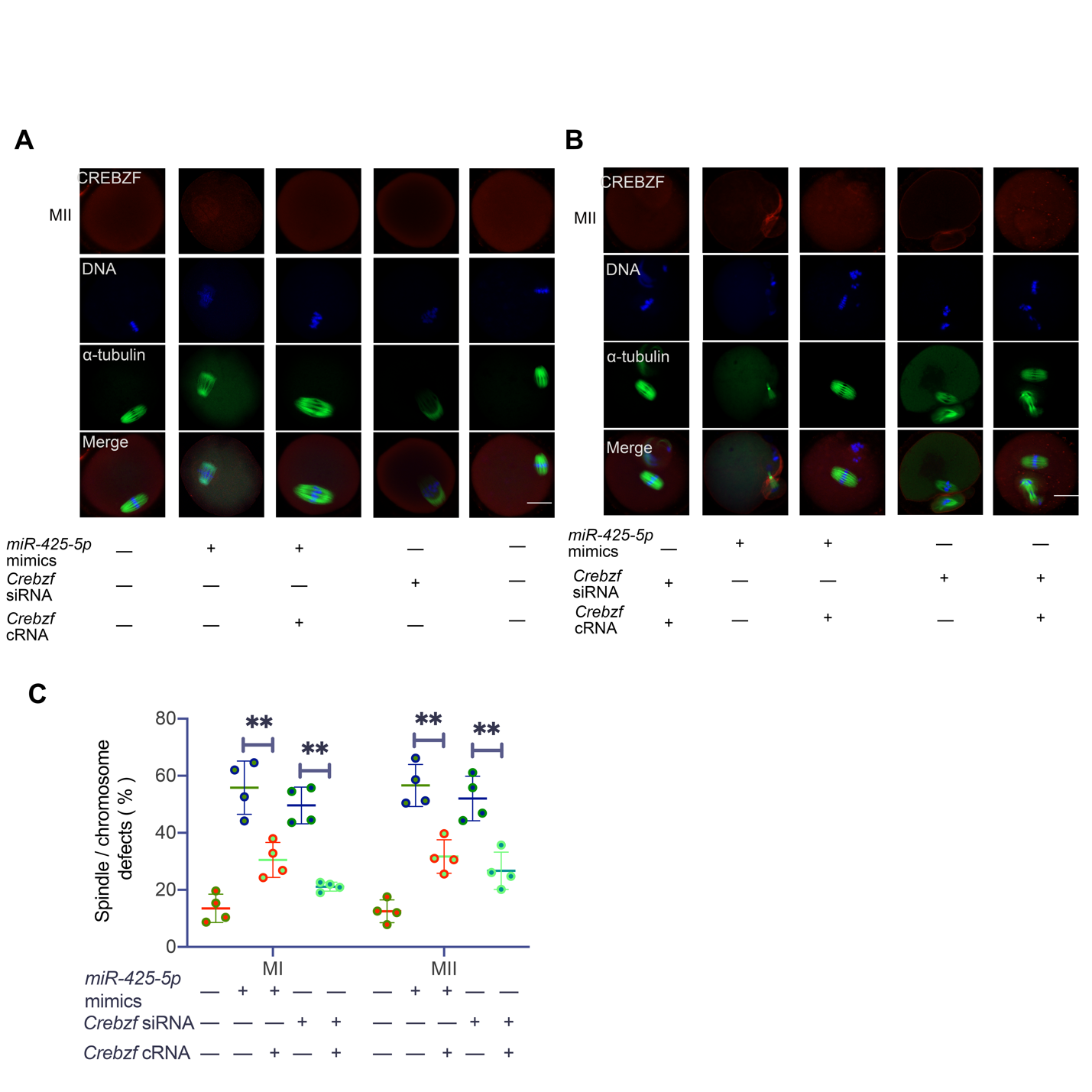
**

**Fig. S4.** Representative images of spindle assembly and chromosome alignment at the Metaphase I (MI) **(A)** and Metaphase II (MII) **(B)** stages of oocytes after microinjecting with *miR-425-5p* mimics, *miR-425-5p* mimics + *Crebzf* cRNA, *Crebzf* siRNA or *Crebzf* siRNA + *Crebzf* cRNA. Scale bar: 20 μm. **(C)** The proportion of oocytes with abnormal spindle and chromosome morphology at the MI and MII stages of oocytes after microinjecting with *miR-425-5p* mimics, *miR-425-5p* mimics + *Crebzf* cRNA, *Crebzf* siRNA or *Crebzf* siRNA + *Crebzf* cRNA (n=70 to 80 in each group). *P* values were determined by two-way ANOVA. Results were representative of at least three independent experiments. **P* < 0.05 and ***P* < 0.01.

**
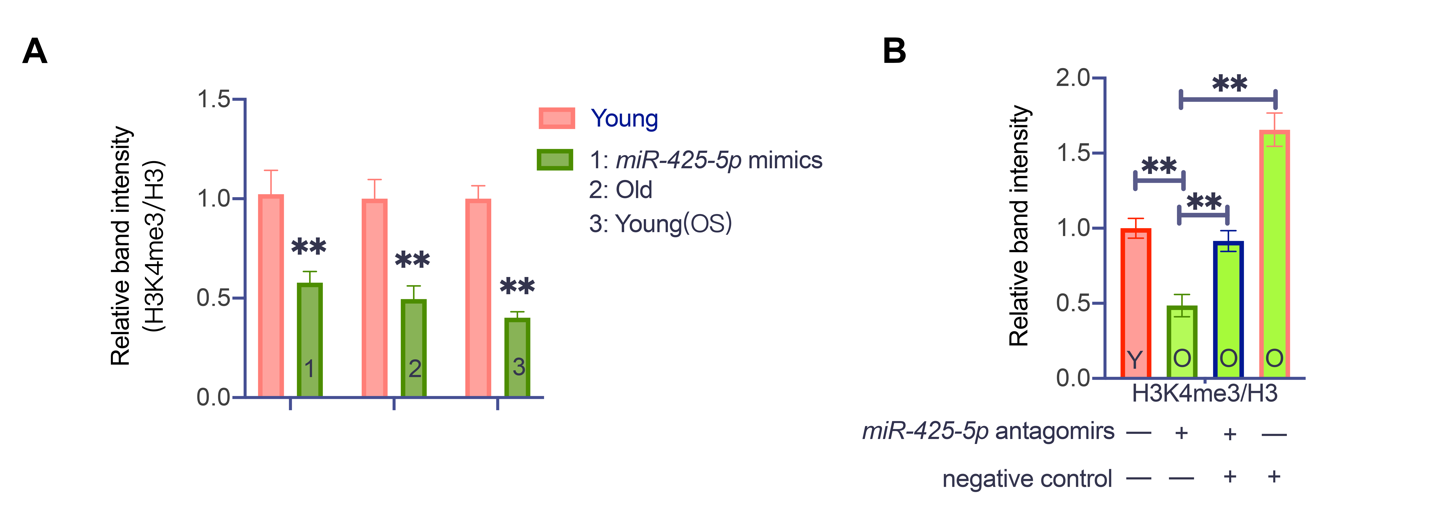
**

**Fig. S5. (A)** Relative band intensity of H3K4me3 after normalization to H3 in NC, *miR-425-5p* mimics and Young（OS） GV1 oocytes. *P* values were determined by multiple T tests. **(B)**: Relative band intensity of H3K4me3 after normalization to H3. Y: Young ; O: Old. *P* values were determined by one-way ANOVA. Results were representative of at least three independent experiments.**P* < 0.05 and ***P* < 0.01.

**Table S1.** Sequences of the primers and siRNAs.

| Gene name | 5′- sequence -3′ |
| --- | --- |
| m-*Crebzf*-F | GCCCGTCTTAATCGGCTCA |
| m-*Crebzf-*R | CGTAGGTAGCGACTCTCCTC |
|  |  |
| m-*Ash1l*-F | CACCATCAAGTCCAGCCTC |
| m-*Ash1l*-R | CCTACTTCCTTTCCCTTTCCAC |
|  |  |
| m-*Setd1a*-F | CCTCCCGGTTCCTAAGTTTAAG |
| m-*Setd1a*-R | CTTCCACCTCTCCATATTTCCG |
|  |  |
| m-*Ash2l*-F | GAAGAGCCTAAAACAGACCCC |
| m-*Ash2l*-R | TGAGATGGCTGGGAAGTAAAC |
|  |  |
| m-*Kmt2d*-F | CCCTTATCTTCCTTTCCCTTCC |
| m-*Kmt2d*-R | AAGCGTCATTTCATCCCCTC |
|  |  |
| m-*Gapdh*-F | GGAGAGTGTTTCCTCGTCCC |
| m-*Gapdh*-R | ATGAAGGGGTCGTTGATGGC |
|  |  |
| si-m-*Crebzf_*001 | GCGTCGTCGTCTCTTAAAA |
| si-m-*Crebzf*_002 | GTCTTAATCGGCTCAAGAA |
| si-m-*Crebzf*_003 | CGGAGGACATGGACTTTCT |

Data S1. (separate file)

Expression of miRNAs in oocytes, sperm and 8-cell embryos from young and reproductively old mice.

Data S2. (separate file)

Target mRNAs of *miR-425-5p* predicted with publicly available databases.
